# Supplementary material for: Three-Dimensional Spheroid Configurations and Cellular Metabolic Properties of Oral Squamous Carcinomas Are Possible Pharmacological and Pathological Indicators
Source: Cancers (Basel). 2023 May 17;15(10):2793. doi: 10.3390/cancers15102793 (PMC10216044; doi:10.3390/cancers15102793)
Supplement: Supplementary file 1 [file cancers-15-02793-s001.zip › cancers-2382121-supplementary.pdf]

Supplementary file
Table S1

KRAS	Taqman	TGCCCTACATCTTATTTCCTCAG	CCTACTGTCGCTAATGGATTGG	/56-FAM/AGGTGGTGG/ZEN/CTGATGCTTTGAACA/3IABkFQ/	NM_033360	6-6
SOX2	Taqman	GTACAACTCCATGACCAGCTC	CTTGACCACGAACCCAT	/56-FAM/CACCTACAG/ZEN/CATGTCCTACTCGCA/3IABkFQ/	NM_003106	1-1
MITF	Taqman	CTCACCATCAGCAACTCCTG	GATTGTCCTTTTTCTGCCTCTC	/56-FAM/AGCTCACAG/ZEN/CGTGTATTTTTCCCACA/3IABkFQ/	NM_000248	11-12a
COL4	Taqman	TGAGTCAGGCTTCATTATGTTCT	AGAGAGGAGCGAGATGTTCA	/56-FAM/TCATACAGA/ZEN/CTTGGCAGCGGCT/3IABkFQ/	NM_001845	51-52
COL6	Taqman	GTGAGGCCTTGGATGATCTC	CCTCGTGGACAAAGTCAAGT	/56-FAM/CAGGTTTCG/ZEN/GTCACAGCGGTAGT/3IABkFQ/	NM_001848	2-3
FN1	Taqman	ACCAATCTTGTAGGACTGACC	CGTCCTAAAGACTCCATGATCTG	/56-FAM/TACAGCTTA/ZEN/TTCTCCCTCGCCCAG/3IABkFQ/	NM_212482	3-4
PGC1a	Taqman	GAGTCTGTTATGGAGTGACATCG	TGTCTGTATCCAAGTCGTTCAC	/56-FAM/ACCAGCCTC/ZEN/TTTGCCCAGATCTTC/3IABkFQ/	NM_013261	1-2
